# The effect of continuity of care on medical costs in patients with chronic shoulder pain

**DOI:** 10.1038/s41598-021-83596-0

**Published:** 2021-02-18

**Authors:** Ju-hyun Oh, Boyoung Jung, Eun-San Kim, Namkwen Kim, In-Hyuk Ha

**Affiliations:** 1 Jaseng Hospital of Korean Medicine, 536 Gangnam-daero, Gangnam-gu, Seoul, 06110 Republic of Korea; 2 Jaseng Spine and Joint Research Institute, Jaseng Medical Foundation, 3F, 538 Gangnam-daero, Gangnam-gu, Seoul, 06110 Republic of Korea; 3 Department of Health Administration, Hanyang Women’s University, 200, Salgoji-gil, Seongdong-gu, Seoul, Republic of Korea; 4 Center for Comparative Effectiveness Research and Economic Evaluation in Korean Medicine, Pusan National University, Yangsan, Gyeongnam, South Korea

**Keywords:** Health care, Disease prevention, Health care economics, Health policy, Health services, Public health

## Abstract

Unnecessary surgery could be prevented through continuity of care (COC). The present study aimed to investigate the relationships between COC, surgery and cost associated with chronic shoulder pain. We used the Health Insurance Review and Assessment Service national patient sample (HIRA-NPS) in 2017. A total of 1717 patients were included. Bice–Boxerman Continuity of Care Index was used as the indicator for measuring the COC. Occurrence of surgery, associated costs, and direct medical costs were analysed. Logistic regression, a two-part model with recycled predictions and generalized linear model with gamma distribution were used. The majority of patients were 40–65 years old (high COC: 68.4%; low COC: 64.4%). The odds ratio (OR) for surgery was 0.41 in the high-COC group compared to the low COC group (95% CI, 0.20 to 0.84). Direct medical cost was 14.09% (95% CI, 8.12% to 19.66%) and 58.00% lower in surgery cost (95% CI, 57.95 to 58.05) in the high-COC group. Interaction with COC and shoulder impingement syndrome was significant lower in direct medical cost (15.05% [95% CI, 1.81% to 26.51%]). High COC was associated with low medical cost in patients diagnosed with chronic shoulder pain.

## Introduction

The number of patients with shoulder pain is increasing at an average rate of 4.6% per year, while the total treatment cost is increasing at a rate of 13.3% per year. Moreover, rotator cuff repair was the most common surgery among patients with shoulder pain in 2014^1^. Shoulder pain can limit work, housework, and participation in leisure activities, which can cause a significant decline in quality of life (QoL)^2^. Especially, chronic shoulder pain has a poor prognosis^3^, and chronic cases after an acute injury are vulnerable to recurrence of dislocation^4^. If there is no improvement in pain, surgery, such as rotator cuff repair and shoulder arthroplasty, may be considered^5^, which could become a major financial burden for the patient^6^. However, the trend in shoulder surgeries such as rotator cuff repair^7^ and shoulder arthroplasty^8^ is increasing.

Continuity of care (COC) is associated with the relationship between each patient and his or her physician^9^. COC is a comprehensive concept, and continuity in treatment experience is associated with patient satisfaction with respect to interpersonal and coordinating aspects of care^9^. The most important aspect of COC is the care delivered over time to the individual patient^10^.

Previous studies have mostly reported on COC and patient health outcomes in cases involving chronic diseases^11–13^. Kim et al.^11^ concluded that continued utilisation of outpatient care could reduce hospitalisation, mortality, and medical costs among diabetic patients. Koopman et al.^12^ found that patients who have a usual provider of care are less likely to have undetected diabetes. Hussey PS et al.^13^ reported that patients with chronic diseases (congestive heart failure, chronic obstructive pulmonary disease, and diabetes) who have high COC showed decreased medical costs, hospitalisation rate, emergency department (ED) use, and complications. Health outcomes in previous studies included length of hospital stay^14–16^ medical cost^11,13,15,17^, patient satisfaction^16^, and ED use^16,18^. However, studies on the relationship between COC and surgery are scarce.

Most COC studies are related to chronic internal medical diseases, such as metabolic syndrome, while very few studies have investigated the relationship between musculoskeletal pain disorders and COC. Jung et al.^15^ concluded that high COC might decrease hospital admission and medical costs for knee osteoarthritis patients. However, to our knowledge, there have been no reports of studies that investigated the effects of COC on health outcomes in patients with shoulder pain. To fill this gap, the present study aimed to investigate the relationships between COC and occurrence of surgery and costs in patients with chronic shoulder pain.

## Method

### Data source

We used the 2017 Korean Health Insurance Review & Assessment Service National Patient Sample (HIRA-NPS) dataset. The HIRA-NPS was composed by a stratified sampling of 3% of all patients using medical services over a 1-year period (approximately 1.45 million patients). The health insurance claims data are nationally representative data from a singular health insurance program operated by the government. All citizens and medical institutions in Korea are registered in national health insurance. HIRA-NPS assures high validity in its representativeness through stratified sampling and protect personal privacy by removing information that could identify each individuals^19^.

Data from the 2017 HIRA-NPS included 285,822,807 cases of medical utilisation by 1,473,083 people. Medical utilisation data contain basic demographic information, such as gender, age, and insurance type, as well as information about the main diagnosis, secondary diagnoses, care provided on outpatient and inpatient basis, all drug information, and status of nursing facility used. Diagnoses are coded according to the International Classification of Disease, Tenth Revision (ICD-10).

### Study population

**Figure 1:**
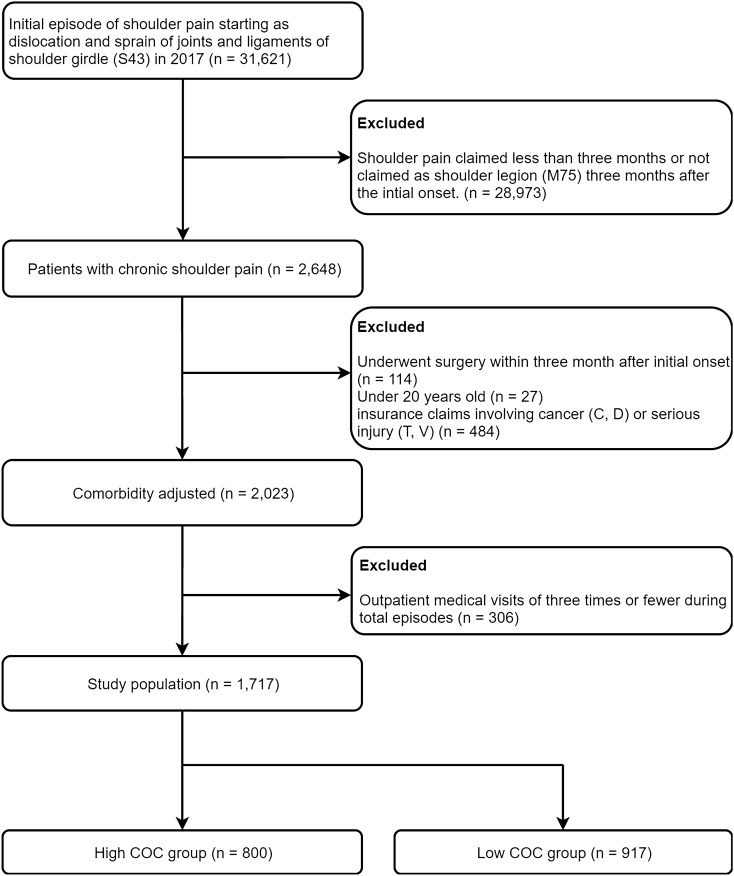
Flowchart of study population. COC: Continuity of Care Index. Values are presented as numbers (percentages) unless otherwise indicated.

The study population consisted of patients who developed chronic shoulder pain after suffering an acute shoulder injury. To select patients with acute shoulder injury, 31,621 patients whose initial episode of shoulder pain started as dislocation and sprain of joints and ligaments of shoulder girdle (S43) was selected. The period of progression into a chronic disease was defined as three months after the initial onset^20^. Accordingly, if shoulder lesion (M75) was claimed as main diagnosis after the initial claim of S43, the patients was considered progressed to chronic shoulder pain (n = 2648). Patients who underwent shoulder surgery during acute phase (i.e. within three months after initial claim of S43) (n = 114) were excluded. Patients aged < 20 years (n = 27) were excluded. Patients diagnosed as red flags such as cancer (C, D) or serious injury (T, V) (n = 484) were excluded. If the number of visits is too low during the overall period of the episodes, the measurement of COC become unstable^21^. Accordingly, previous studies usually included only patients with outpatient medical visits of four or more times during total episodes^14,22,23^. The present study applied the same criterion and excluded patients with outpatient medical visits of three times or fewer during total episodes (n = 306). As a result, 1717 patients were included in the final analysis (Fig. 1).

### Continuity of care

The present study used the Bice-Boxerman COC index^24^ as the index for measuring COC. COC ranges between 0 and 1, with a higher value indicating higher COC. COC, which is a frequently used index to measure continuity^25^, accounts for variance of service providers that a single patient visited during the total episodes and concentration of visits for a specific service provider (Eq. 1). In the process of calculating COC, utilisation of outpatient care for main diagnosis of shoulder pain (S43, M75) was used. High- and low-COC groups were divided based on median values. To our best knowledge, there is no absolute standard for differentiating high and low COC, and as a result, previous studies applied various standards according to the research model, including the median^26^, tertiles^27^, quartiles^15^, and continuous^13^. Due to the relatively fewer events as compared to previous studies, we used the median as the standard for differentiating high and low COC in the main study. Other standard was used for sensitivity analysis.

### Equation 1: the equation of Bice-Boxerman continuity of care index

1 $$COC=\frac{{\sum }_{i=1}^{M}{n}_{i}^{2}-N}{N(N-1)}$$

M: The number of providers; n_i_: The number of visits to provider I; N: Total number of visits during episode.

### Outcome

The dependent variables included occurrence of surgery, conservative treatments, surgery associated cost, and total medical cost. Occurrence of surgery was defined as possible surgery due to failed care of chronic shoulder surgery.

Surgical intervention must be chosen when there is no improvement or exacerbation of symptoms during rehabilitation^28^. Typical diseases of this type include those that involve the rotator cuff, such as shoulder impingement syndrome and rotator cuff tear. Surgical intervention includes acromioplasty and suturing if rotator cuff tear is involved^28^. Also, Partial or total arthroplasty is needed if open reduction is not possible due to inadequate fixation of the humeral head or humeral head wear due to serious injury or deterioration^28^. Accordingly, among the possible surgical interventions after an acute sprain, acromioplasty, rotator cuff repair (N0935, N0936, N0937, N0938), and shoulder arthroplasty (N2071, N2711) are defined as *events*^29^*.* Conservative treatments patients received during the episodes includes physical therapy, X-ray, injection, acupuncture, nerve block, non-steroidal anti-inflammatory drugs, paracetamol, muscle relaxants, corticosteroids and opioids. The details of conservative treatments are described in supplementary table S1.

Regarding medical costs, total claimed medical costs from the national health insurance claims data were analysed. Medical costs were divided into direct and indirect medical costs. Direct medical costs include costs associated with outpatient care, inpatient care, ED use, medication, diagnosis, treatment, medical devices, and hospital stay. Indirect medical costs include loss of productivity due to a disease and associated costs^30^. Only direct medical costs associated with medical utilisation were included as medical costs^31^ while total medical costs claimed for shoulder pain during patient episodes were analysed. All costs was converted to USD with currency status in 31th June 2017 (1 USD = 1145 KRW).

### Covariates

Gender, age, and insurance type were included as sociodemographic characteristics. Age groups were divided based on 40 and 65 years according to the definition of life transition periods in Korea. To adjust for severity, adhesive capsulitis of the shoulder (ACS; ICD-10: M750) and shoulder impingement syndrome (SIS; ICD-10: M754), which have the highest prevalence among chronic shoulder diseases in Korea, were used as comorbidities. The Charlson Comorbidity Index (CCI) was also used^32^. The CCI is most widely used index and also used for musculoskeletal disease^33^. The CCI was calculated with presences of 17 comorbidities. Presence of comorbidity was examined based on whether those disease codes were diagnosed before the end of the episodes. Moreover, the total number of medical visits for shoulder pain during the episodes was adjusted as previous studies^13^, assuming that more severe patients would be more likely to use medical services. It was considered that the characteristics of the main attending medical institution would affect continuity and patient behaviour, and thus, the institution that the patient had visited the most during the total episode was defined as the primary provider and accounted for whether the institution is a clinic or hospital^11,34,35^. A total of 405 patients had two or more medical institutions most frequently visited with the same number of visits (e.g., total of 15 visits with 5 visits each to institution A, B and C). In this case, one institution was selected randomly.

### Statistical analysis

The difference in basic characteristics between groups was examined with independent t-test and chi-square test. To analyse the effects of COC on surgery, a multivariate logistic regression analysis was performed with covariates controlled. However, a quasi-complete problem occurred in the logistic regression analysis due to the relatively small number of events (n = 41). Since this made it impossible to estimate the maximum likelihood, we performed Firth’s logistic regression analysis^36^.

Two types of costs were analysed. First, the effects of COC on medical costs during the total episodes were analysed. The distribution of medical costs was right-skewed, and as a result, heteroscedasticity existed in the residuals. To resolve this, generalised linear model (GLM) with gamma distribution and log link function were used^37^. The difference in cost was expressed as percentage reduction due to high COC. For this, the low-COC was referenced, coefficients were exponentiated, multiplied by 100, and subtracted from 100 to analyse the effects of COC on surgery cost, a two-part model was employed first^38^. The two-part model was used because most patients did not undergo surgery, and therefore, their surgery cost was 0. As the first part, multivariate logistic regression for analysing the effects of COC on surgery was performed. With estimated model, the probability of undergoing surgery was predicted for each patients. As the second part, GLM with gamma distribution was performed only on patients who underwent surgery. Based on the estimated model, the surgery cost of the entire study population was predicted. Subsequently, the predicted probability and surgery costs in each part were multiplied. The second part was analysed only if the effects of COC was statistically significant (*p* < 0.05) in the first part, because if the effect of COC on surgery was not significant, the analysis on the effects of COC on surgery costs would be meaningless.

Next, recycled predictions were performed to analyse the marginal effects of COC on surgery cost^39^. With two-part model estimated previously, the costs of surgery were predicted with two scenario, all patients with low COC scenario and high COC scenario. The other covariates were fixed. Then, differences in surgery costs between each scenario were calculated. The mean and the 95% confidence interval was estimated with 1000 bootstrapping.

However, using data from patients who underwent surgery to predict the cost of other patients may lead to biased results. If generalised linear model is adopted under such situations, over-fitting may occur due to the number of patients being too low. Following the discussion by Kogure^40^, we performed a Lasso regression analysis^41^. With this, L1 penalty is applied when estimating the coefficient by ordinary least squares as a trade-off between bias and variance. Kogure^40^ demonstrated that the accuracy of prediction could be increased by regularisation through a penalised model in the two-part model. In sensitivity analysis, a model including basic demographic characteristics and severity, ridge regression^42^, and elastic net^43^ were performed, and the results were compared. Lasso, ridge regression, and elastic net were performed with y log normalised, and Duan’s smearing factor was added in the process of deriving the exponential.

For the sensitivity analysis, we first used the tertiles as the standard for differentiating high, middle and low COC. Due to the very small of number of the events, two-part model was not performed. Second, we assumed situations where the act of insurance claim may have been inaccurate. According to Park^44^, the accuracy of insurance claims in Korea is approximately 80%. We assumed that the act of insurance claims most likely to be inaccurate was from patients who developed chronic shoulder pain (M75) as acute pain (S43). Accordingly, the same process was carried out after randomly including 20% patients among those who claimed S43 even three months after the onset of acute shoulder injury. Moreover, subgroup analyses were performed based on the presence of chronic diseases including frozen shoulder and shoulder impingement syndrome, and the interaction effect between COC and each disease was derived. When analysing the medical costs, the number of visits was considered to be important. Accordingly, the covariates were included in the model hierarchically, and we observed changes in the coefficient of COC each time a covariate was added. SAS 9.4 and R studio version 1.1.463 was used for statistical analysis.

## Results

**Figure 2:**
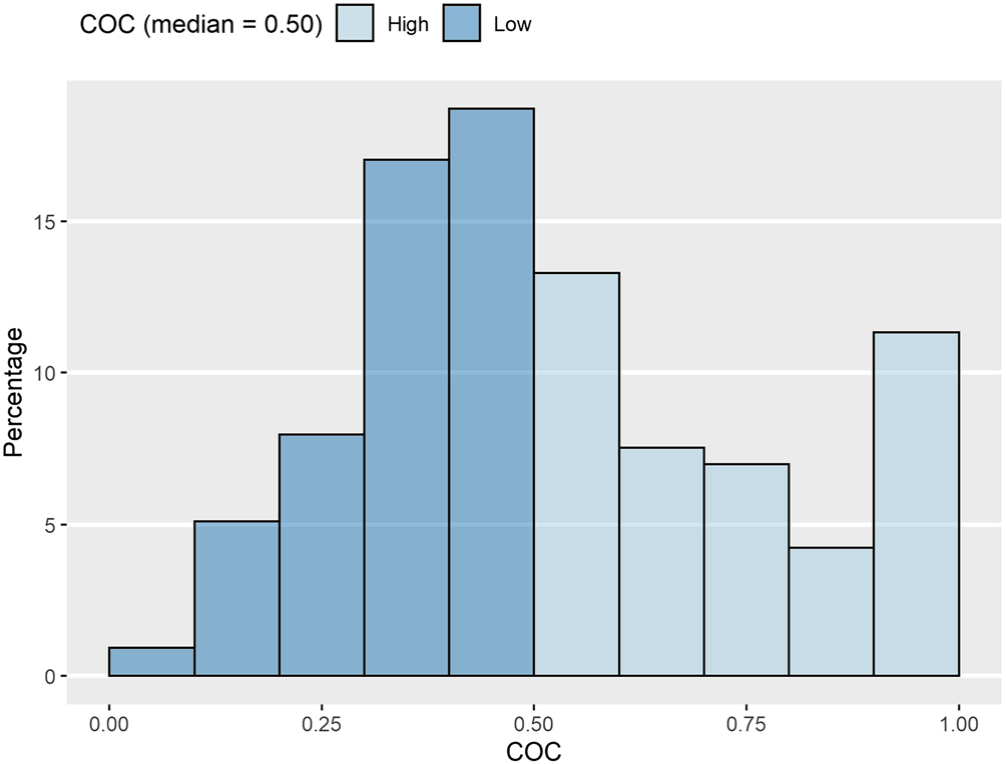
Distribution of COC. COC: Continuity of Care Index. The median of COC distribution was used for criteria.

The basic patient demographics are shown in Table 1. The patients were divided into high- and low-COC groups based on the median COC value of 0.50 and were then compared. The overall distribution of COC is shown in Fig. 2. With respect to basic demographics, their distribution was similar between the high- and low-COC groups. However, the low-COC group had a higher prevalence of shoulder impingement syndrome (high COC: 25.5%; low COC: 33.4%), while the high-COC group had a higher total number of medical visits (high COC: mean = 16.1 ± 16.1, median = 11; low COC: mean = 12.1 ± 10.9, median = 9).

**Table 1:** Basic characteristics of patients.

	COC (median = 0.50)		
	High (n = 800)	Low (n = 917)	*P*-value
**Age**			0.212
21–40	73 (9.1)	99 (10.8)	
40–65	547 (68.4)	591 (64.4)	
65–	180 (22.5)	227 (24.8)	
**Gender**			0.394
Female	418 (52.3)	498 (45.7)	
Male	382 (47.8)	419 (54.3)	
**Coverage type**			0.341
National Health Insurance	763 (95.4)	883 (96.3)	
Beneficiary	37 (4.6)	34 (3.7)	
**Frozen shoulder**			0.001
Yes	370 (46.3)	496 (45.9)	
No	430 (53.8)	421 (54.1)	
**Shoulder impingement syndrome**			< 0.001
Yes	204 (25.5)	306 (33.4)	
No	596 (74.5)	611 (66.6)	
**Charlson comorbidity index**			0.658
0	245 (30.6)	270 (29.4)	
1	202 (25.3)	215 (23.4)	
2	92 (11.5)	113 (12.8)	
3 +	261 (32.6)	319 (34.8)	
**Primary provider type**			0.030
Clinic	691 (86.4)	757 (82.6)	
Hospital	109 (13.6)	160 (17.4)	
**The total number of medical visits**			< 0.001
Mean ± SD	16.1 ± 16.1	12.1 ± 10.9	
Median	11	9	

Values are presented as numbers (column percentages) for categorical variables and mean ± standard deviation (SD) for continuous variable. To examine difference between groups, the independent t test was used for continuous variables and chi-square test was used for categorical variables.

*COC* Continuity of Care Index.

**Figure 3:**
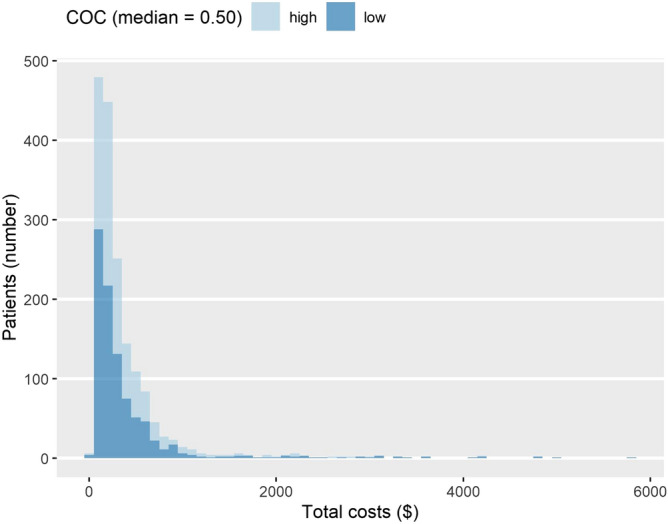
The distribution of total medical costs. COC: Continuity of Care Index. The medical costs are calculated in United States dollar (20170631).

**Figure 4:**
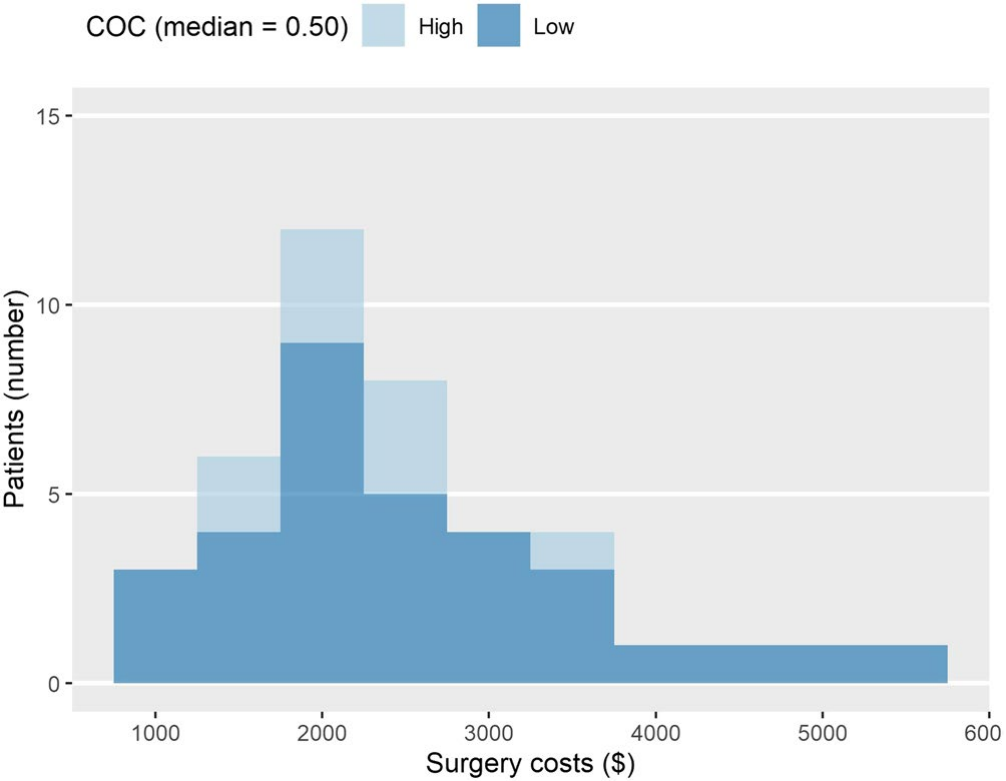
The distribution of surgery costs. COC: Continuity of Care Index. Surgery costs are calculated only for patients who underwent shoulder surgery (n = 41). The medical costs are calculated in United States dollars (20170631).

Table 2 describes the type of shoulder surgery and associated costs for the high- and low-COC groups. There were 41 surgery cases, most of which were to repair a rotator cuff tear (n = 40). The number of surgeries was higher in the low-COC group (high COC: n = 9; low COC: n = 32), with a noteworthy difference in incidence between the high COC (11.25 per 1000 population) and low COC (34.89 per 1000 population) groups. With respect to total medical cost, the median was higher and mean was lower in the high-COC group than in the low-COC group (high COC: mean = $364.43 ± 401.36, median = $236.24; low COC: mean = $396.52 ± 602.08, median = $220.99). A similar tendency was found for surgery cost. The distributions of total medical cost and surgery cost are shown in Figs. 3 and 4. The details of conservative treatments each group received during the episodes were described in Supplementary Table 1. In brief, low-COC group had more nerve block, and medications such as corticosteroids and opioids.

**Table 2:** Types of surgery and medical costs by COC.

	COC (median = 0.50)	
	High (n = 800)	Low (n = 917)
**Occurrence of surgery**		
Rotator cuff surgery	9	31
Arthroplasty	0	1
Total incidence rate		
(per 1000 population)	11.25	34.89
**Medical costs, median**		
Total direct medical costs	236.24	220.99
Direct medical costs per visit	19.88	23.58
Surgery costs	2228.82	2254.59
**Medical costs, mean ± SD**		
Total direct medical costs	364.43 ± 401.36	396.52 ± 602.08
Direct medical costs per visit	25.86 ± 31.05	35.63 ± 59.19
Surgery costs	2255.02 ± 562.76	2510.21 ± 1110.28

The occurrence of surgery is presented with numbers and incidence per 1000 population.

Surgery costs are calculated only for patients who underwent shoulder surgery (n = 41).

The medical costs are calculated in United States dollars in 31th June 2017 (1 USD = 1145 KRW).

In the analysis of the effects of COC on surgery and medical costs (Table 3), odds ratio (OR) for surgery was lower by 0.41-fold in the high-COC group than in the low COC group (95% CI 0.20 to 0.84). The results showed a tendency of 14.09% less per person in direct medical costs (95% CI, 8.12% to 19.66%) and 58.00% less in surgery cost (95% CI, 57.95% to 58.05%). Analyses with other prediction models for surgery costs showed that the tendency was consistent (Supplementary Table S2).

**Table 3:** The effects of COC on surgery and medical costs.

	Unadjusted	Adjusted
Surgery (OR)	0.33 (0.16 to 0.68)	0.41 (0.20 to 0.84)
**Medical costs (lower)**		
Direct medical costs (%)	8.09 (0.38 to 15.2)	14.09 (8.12 to 19.66)
Surgery costs (%)	58.00 (57.95 to 58.05)	

The reference group is low COC group (COC < 0.50 [median]). Effects of COC on surgery cost was estimated using a two-part model. Adjusted covariates are age in category, Gender, Coverage type, frozen shoulder, shoulder impingement syndrome, Charlson comorbidity index, primary provider type, and the total number of medical visits.

In the subgroup analyses according to presence of chronic disease, the results showed that the effects of COC on surgery in patients with chronic disease were not significant (frozen shoulder OR: 0.53 [95% CI, 0.21 to 1.32], shoulder impingement syndrome OR: 0.53 [95% CI, 0.23 to 1.20]). Interaction was not significant either (frozen shoulder OR: 1.66 [95% CI, 0.38 to 7.54]; shoulder impingement syndrome OR: 1.59 [95% CI, 0.33 to 9.91]). However, the effects of COC on medical cost were significant, and interaction appeared in the direction of reducing medical costs in patients with shoulder impingement syndrome (15.05% [95% CI, 1.81% to 26.51%]) (Table 4).

**Table 4:** Subgroup analysis with comorbidity in shoulder disease.

	Patients with chronic disease		Patients without chronic disease		Interaction
	Unadjusted	Adjusted	Unadjusted	Adjusted	
**Frozen shoulder (n = 866)**					
Surgery (OR)	0.39 (0.15 to 1.02)	0.53 (0.21 to 1.32)	0.30 (0.10 to 0.86)	0.32 (0.12 to 0.87)	1.66 (0.38 to 7.54)
**Medical costs (lower)**					
Direct medical costs (%)	8.41 (− 2.69 to 18.31)	13.90 (5.16 to 21.84)	3.30 (− 8.14 to 13.53)	15.79 (7.63 to 23.23)	− 0.32 (− 14.47 to 12.08)
Surgery cost (%)	–		73.40 (73.33 to 73.46)		
**Shoulder impingement syndrome (n = 510)**					
Surgery (OR)	0.46 (0.20 to 1.07)	0.53 (0.23 to 1.20)	0.27 (0.07 to 1.08)	0.28 (0.08 to 0.94)	1.59 (0.33 to 9.91)
**Medical costs (lower)**					
Direct medical costs (%)	16.31 (1.56 to 28.85)	20.01 (6.86 to 31.3)	− 3.63 (− 13.23 to 5.15)	11.23 (4.96 to 17.08)	15.05 (1.81 to 26.51)
Surgery cost (%)	–		82.35 (82.33 to 82.37)		

The reference group is low COC group (COC < 0.50 [median]). Effects of COC on surgery cost was estimated using a two-part model. Adjusted covariates are age in category, Gender, Coverage type, frozen shoulder, shoulder impingement syndrome, Charlson comorbidity index, primary provider type, and the total number of medical visits. The interaction between comorbidities and COC was presented. Dashed means reduction in surgery costs was not estimated, because the effect of COC on surgery was not significant.

In the analysis with tertiles, OR for surgery was lower by 0.40-fold in the middle-COC group, however, the OR was not significant in the high-COC group (middle-COC OR: 0.40 [95% CI, 0.19 to 0.85]; high-COC OR: 0.51 [95% CI, 0.23 to 1.12]). P-trend was significant (*p* = 0.038). In medical cost, middle and high-COC group had lower medical cost than low-COC group and the P-trend was significant (middle-COC: 12.25% [95% CI, 4.90% to 19.05%], high-COC: 15.93% [95% CI, 8.64% to 22.63%]); P-trend: *p* < 0.001) (Supplementary Table S3). The tendency in the analysis accounting for inaccurate claims in data was similar to that of the main analysis (Supplementary Table S4). When the covariates were hierarchically included in the model, the largest decrease in AIC and BIC was found when the total number of medical visits was included (decrease in AIC: 635.9957; decrease in BIC: 630.5473) (Supplementary Table S5).

## Discussion

The present study investigated the effects of COC on the treatment cost and surgery among patients with chronic shoulder pain. Among patients in Korea who had filed a national health insurance claim for chronic shoulder pain after acute shoulder injury, a higher COC resulted in lower risk of surgery (OR: 0.41; 95% CI, 0.20 to 0.84), medical cost per visit (14.09%; 95% CI, 8.12% to 19.66%), and surgery cost (58.00%; 95% CI, 57.95% to 58.05%). Such findings had same tendency as previous studies that investigated the effects of COC on health outcomes of patients with chronic diseases^11,13,14,16–18^, in which high COC showed an association with low medical cost. They were also similar to other studies on ED visits^16,18^ in that exacerbation of symptoms was prevented. However, most of these preceding studies involved metabolic disorders, while we were able to find very few articles on musculoskeletal disorders and COC^15^. This article^15^ reported that higher COC resulted in lower hospitalisation and treatment costs among patients with osteoarthritis of the knees. The present study demonstrated that medical cost and risk and cost of surgery for severe cases decreased in patients with chronic shoulder pain.

Subgroup analyses showed that COC effects on the direct medical costs was greater in patients with shoulder impingement syndrome (15.05%; 95% CI, 1.81% to 26.51%). According to a previous study^45^ patients with shoulder impingement syndrome had higher costs associated with procedure, surgery, and hospitalisation than patients with frozen shoulder. It is believed that COC prevented such medical uses, which made the medical cost reduced more significantly than other diseases. However, the high-COC group had a higher number of medical visits and a higher median value for direct medical costs. Such results suggest the need for developing a policy that could reduce the number of medical visits while maintaining a high level of COC.

In caring for chronic shoulder disease after an injury, COC may provide high quality care in conservative treatment. Most cases of shoulder pain are primarily treated by conservative treatment. For example, rotator cuff tear or impingement syndrome with mild severity could be improved with at least three months of conservative treatment, such as rehabilitation therapy including muscle strengthening exercises^28^. During the process of taking part in a repetitive rehabilitation program, forming a rapport between the patient and physician could be helpful. According to the previous study, COC could be helpful in forming such rapport^46^.

The present study had some limitations. Firstly, the number of events was too small. In the two-part model prediction, the number of surgery cases was low at only 41. To compensate for this limitation, penalised regression and multiple models were used. The results showed similar tendencies, but because of the small number of events, a validation set for LASSO regression could not be created. Second, due to the limitation of the raw data (e.g., claims data), only a cross-sectional study with one-year observational period of 2017 could be conducted. Due to the short follow-up period, the analysis might be restricted to the effect of COC in the early phase of chronic shoulder pain. In future studies, it would be necessary to establish a long-term cohort to investigate the prognosis of chronic diseases. Third, in the analysis with tertiles standard, the dose–response was unclear in the OR for surgery. But the total cases of surgery was very small and dividing the groups into tertiles could dramatically lower the power of the logistic regression. The further analysis should be conducted with more samples. Fourth, although sensitivity analysis was performed, accuracy of claims data might be problematic^47^. However, considering that previous studies reported that the accuracy of diagnosis in insurance claims data is 80%, the sensitivity analysis showed a similar tendency with the main results. Lastly, because only insurance claims data for the shoulder were analysed, we could not predict all costs associated with the shoulder. Generally, total medical costs are categorised as direct, indirect, and non-medical costs^48^. The present study estimated only the direct medical costs covered by insurance. Non-medical costs and indirect cost should be considered in the further studies.

Despite the limitations, the present study has potential importance in that it used health insurance claims data most representative of the general population of Korea to investigate samples of patients with shoulder disease. We confirmed robustness through various sensitivity analyses, and the heterogeneity of outcome according to patient characteristics was analysed by subgroup analyses. Moreover, to our best knowledge, the present study is the first study that examines the relationship between COC and shoulder disease. Therefore, the findings in the present study could provide meaningful and useful information to clinicians, policy makers, and patients who use medical services for shoulder pain.

## Supplementary Information

Supplementary Information.

## Data Availability

The HIRA-NPS is provided by the Health Insurance Service & Assessment Service in Korea. To protect privacy, access to the data is available only for certified researchers in South Korea. The study protocol was approved by the Institutional Review Board of Jaseng Hospital of Korean Medicine (JASENG 2019-07-005) and followed relevant guidelines. The requirement of informed consent from the study population was waived by the same IRB.

## References

[1] Health Insurance Review and Assessment Service. *Press release about trends of shoulder disease in 2010–2014*. http://www.hira.or.kr/bbsDummy.do?pgmid=HIRAA020041000100&brdScnBltNo=4&brdBltNo=8940 (2015). Accessed 4 Jan 2021.

[2] Council NR (2001). Musculoskeletal Disorders and the Workplace: Low Back and Upper Extremities.

[3] Reilingh ML, Kuijpers T, Tanja-Harfterkamp AM, Van der Windt DA (2008). Course and prognosis of shoulder symptoms in general practice. Rheumatology (Oxford).

[4] Eljabu W, Klinger HM, von Knoch M (2017). The natural course of shoulder instability and treatment trends: a systematic review. J. Orthop. Trauma.

[5] Burbank KM, Stevenson JH, Czarnecki GR, Dorfman J (2008). Chronic shoulder pain: part II Treatment. Am. Fam. Phys..

[6] Black EM, Higgins LD, Warner JJ (2013). Value-based shoulder surgery: practicing outcomes-driven, cost-conscious care. J. Shoulder Elbow Surg..

[7] Colvin AC, Egorova N, Harrison AK, Moskowitz A, Flatow EL (2012). National trends in rotator cuff repair. J. Shoulder Elbow Surg..

[8] Kim SH, Wise BL, Zhang Y, Szabo RM (2011). Increasing incidence of shoulder arthroplasty in the United States. J. Bone Jt. Surg..

[9] Gulliford M, Naithani S, Morgan M (2006). What is 'continuity of care'?. J. Health Serv. Res. Policy.

[10] Haggerty JL, Reid RJ, Freeman GK, Starfield BH, Adair CE, McKendry R (2003). Continuity of care: a multidisciplinary review. BMJ.

[11] Kim J, Kim H, Kim H, Min KW, Park SW, Park IB (2006). Current status of the continuity of ambulatory diabetes care and its impact on health outcomes and medical cost in Korea using national health insurance database. J. Korean Diabetes.

[12] Koopman RJ, Mainous AG, Baker R, Gill JM, Gilbert GE (2003). Continuity of care and recognition of diabetes, hypertension, and hypercholesterolemia. Arch. Intern. Med..

[13] Hussey PS, Schneider EC, Rudin RS, Fox DS, Lai J, Pollack CE (2014). Continuity and the costs of care for chronic disease. JAMA Intern. Med..

[14] Nyweide DJ, Anthony DL, Bynum JP, Strawderman RL, Weeks WB, Casalino LP, Fisher ES (2013). Continuity of care and the risk of preventable hospitalization in older adults. JAMA Intern. Med..

[15] Jung B, Cho KH, Lee DH, Kim S (2018). The effects of continuity of care on hospital utilization in patients with knee osteoarthritis: analysis of Nationwide insurance data. BMC Health Serv. Res..

[16] Cabana MD, Jee SH (2004). Does continuity of care improve patient outcomes. Fam. Pract..

[17] De Maeseneer JM, De Prins L, Gosset C, Heyerick J (2003). Provider continuity in family medicine: does it make a difference for total health care costs?. Ann. Fam. Med..

[18] Gill JM, Mainous AG, Nsereko M (2000). The effect of continuity of care on emergency department use. Arch. Fam. Med..

[19] 19Kim, R. Y. Introduction of HIRA national patient sample (HIRA-NPS) data. 37–47 (2012).

[20] Hegmann KT (2016). Shoulder disorder guideline.

[21] Christakis DA, Wright JA, Koepsell TD, Emerson S, Connell FA (1999). Is greater continuity of care associated with less emergency department utilization?. Pediatrics.

[22] Amjad H, Carmichael D, Austin AM, Chang CH, Bynum JP (2016). Continuity of care and health care utilization in older adults with dementia in fee-for-service Medicare. JAMA Intern. Med..

[23] Christakis DA, Mell L, Koepsell TD, Zimmerman FJ, Connell FA (2001). Association of lower continuity of care with greater risk of emergency department use and hospitalization in children. Pediatrics.

[24] Bice TW, Boxerman SB (1977). A quantitative measure of continuity of care. Med. Care.

[25] Saultz JW (2003). Defining and measuring interpersonal continuity of care. Ann. Fam. Med..

[26] Shin DW, Cho J, Yang HK, Park JH, Lee H, Kim H (2014). Impact of continuity of care on mortality and health care costs: a nationwide cohort study in Korea. Ann. Fam. Med..

[27] Barker I, Steventon A, Deeny SR (2017). Association between continuity of care in general practice and hospital admissions for ambulatory care sensitive conditions: cross sectional study of routinely collected, person level data. BMJ.

[28] Lee YG (2004). Surgical treatment of shoulder pain. Clin. Pain.

[29] Health Insurance Review and Assessment Service. Standard guide for calculating disease/practice statistics. 245,248 (2018).

[30] Rat AC, Boissier MC (2004). Rheumatoid arthritis: direct and indirect costs. Jt. Bone Spine.

[31] Eisenberg JM (1989). Clinical economics: a guide to the economic analysis of clinical practices. JAMA.

[32] Charlson ME, Pompei P, Ales KL, MacKenzie CR (1987). A new method of classifying prognostic comorbidity in longitudinal studies: development and validation. J. Clin. Epidemiol..

[33] Hilton ME, Gioe T, Noorbaloochi S, Singh JA (2016). Increasing comorbidity is associated with worsening physical function and pain after primary total knee arthroplasty. BMC Musculoskelet. Disord..

[34] Cho KH, Lee SG, Jun B, Jung BY, Kim JH, Park EC (2015). Effects of continuity of care on hospital admission in patients with type 2 diabetes: analysis of nationwide insurance data. BMC Health Serv. Res..

[35] Hong JS, Kang HC, Kim J (2010). Continuity of care for elderly patients with diabetes mellitus, hypertension, asthma, and chronic obstructive pulmonary disease in Korea. J. Korean Med. Sci..

[36] Heinze G, Schemper M (2002). A solution to the problem of separation in logistic regression. Stat. Med..

[37] Blough DK, Ramsey SD (2000). Using generalized linear models to assess medical care costs. Health. Serv. Outcome Res. Methods.

[38] Manning WG, Morris CN, Newhouse JP, Orr LL, Duan N, Keeler EB (1981). A two-part model of the demand for medical care: preliminary results from the health insurance study.

[39] Graubard BI, Korn EL (1999). Predictive margins with survey data. Biometrics.

[40] Kogure, A. Predicting Health Care Costs by Two-part Model with Sparse Regularization. The World Risk and Insurance Economics Congress (2015).

[41] Tibshirani R (1996). Regression shrinkage and selection via the lasso. J. R. Stat. Soc. Ser. B Stat. Methodol..

[42] Hoerl AE, Kennard RW (1970). Ridge regression: biased estimation for nonorthogonal problems. Technometrics.

[43] Zou H, Hastie T (2005). Regularization and variable selection via the elastic net. J. R. Stat. Soc. Ser. B Stat. Methodol..

[44] Park, E. C. Assessment and improvement measures for consistency between health insurance claim disease code and medical records (2017).

[45] Joo H, Lee YJ, Shin JS, Lee J, Kim MR, Koh W (2017). Medical service use and usual care of common shoulder disorders in Korea: a cross-sectional study using the Health Insurance Review and Assessment Service National Patient Sample. BMJ Open.

[46] Ettner SL (1999). The relationship between continuity of care and the health behaviors of patients: does having a usual physician make a difference?. Med. Care.

[47] Kim J, Yoon S, Kim LY, Kim DS (2017). Towards actualizing the value potential of Korea Health Insurance Review and Assessment (HIRA) data as a resource for health research: strengths, limitations, applications, and strategies for optimal use of HIRA data. J. Korean Med. Sci..

[48] Hodgson TA, Meiners MR (1982). Cost-of-illness methodology: a guide to current practices and procedures. Milbank Mem. Fund. Q. Health Soc..

