# Supplementary material for: The effect of continuity of care on medical costs in patients with chronic shoulder pain
Source: Sci Rep. 2021 Feb 18;11:4077. doi: 10.1038/s41598-021-83596-0 (PMC7893020; doi:10.1038/s41598-021-83596-0)
Supplement: Supplementary file 1 — Supplementary Information. [file 41598_2021_83596_MOESM1_ESM.docx]

**The Effect of Continuity of Care on Medical Costs in Patients with Chronic Shoulder Pain**

Ju-hyun Oh ^1^, Boyoung Jung ^2, 3^, Eun-San Kim ^2^, Namkwen Kim ^4^, In-Hyuk Ha ^2^*

^1^ Jaseng Hospital of Korean Medicine, 536 Gangnam-daero, Gangnam-gu, Seoul 06110, Republic of Korea;

^2^ Jaseng Spine and Joint Research Institute, Jaseng Medical Foundation, 3F, 538 Gangnam-daero, Gangnam-gu, Seoul 06110, Republic of Korea
^3^ Department of Health Administration, Hanyang Women’s University, 200, Salgoji-gil, Seongdong-gu, Seoul, Republic of Korea

^4^ Center for Comparative Effectiveness Research & Economic Evaluation in Korean Medicine, Pusan National University, Yangsan, Gyeongnam, South Korea

**Corresponding author:**

In-Hyuk Ha

Jaseng Spine and Joint Research Institute, Jaseng Medical Foundation, 3F, 538 Gangnam-daero, Gangnam-gu, Seoul 06110, Republic of Korea

E-mail: [hanihata@gmail.com](mailto:hanihata@gmail.com)

**Supplementary Table S1. The details of conservative treatments patients received during episodes by group**

|  | Number of patients | | Number of Visits | |
| --- | --- | --- | --- | --- |
|  | High-COC | Low-COC | High-COC | Low-COC |
| **Total** | 800 | 917 | 12943 | 11210 |
| **Medical procedures** |  |  |  |  |
| Physical therapy | 527 (65.9) | 664 (72.4) | 5169 (39.9) | 3567 (31.8) |
| X-ray | 453 (56.6) | 626 (68.3) | 636 (4.9) | 3792 (33.8) |
| Injection | 411 (51.4) | 623 (67.9) | 1528 (11.8) | 1034 (9.2) |
| Acupuncture | 405 (50.6) | 533 (58.1) | 4441 (34.3) | 1595 (14.2) |
| Nerve block | 273 (34.1) | 424 (46.2) | 1219 (9.4) | 1481 (13.2) |
| **Prescribed medications** |  |  |  |  |
| NSAID | 621 (77.6) | 772 (84.2) | 3034 (23.4) | 3293 (29.4) |
| Paracetamol | 96 (12) | 130 (14.2) | 225 (1.7) | 253 (2.3) |
| Muscle relaxants | 396 (49.5) | 514 (56.1) | 1632 (12.6) | 1619 (14.4) |
| Corticosteroids | 279 (34.9) | 421 (45.9) | 814 (6.3) | 1038 (9.3) |
| Opioids | 319 (39.9) | 446 (48.6) | 1323 (10.2) | 1328 (11.8) |
| The high and low-COC group was divided by median (COC = 0.5). The total means total number of patients and visits during episodes of chronic shoulder pain.  Physical therapy includes heat therapy, transcutaneous electrical nerve stimulation, interferential Current Therapy and myofascial trigger point injection therapy. Injection includes subcutaneous, intramuscular, intravenous, joint injection and etc. X-ray and nerve block was defined interventions performed on shoulder and related (e.g. axillary and clavicular joint) area.  Prescribed medications includes non-steroidal anti-inflammatory drugs (NSAID; ATC code: M01A), paracetamol (N02BE), muscle relaxants (M03B), corticosteroids (H02) and opioids (N02A). | | | | |

**Supplementary Table S2. Results of surgery costs analysis with other prediction models.**

|  | Predicted with penalised regression | | | Predicted with gamma log-link regression | |
| --- | --- | --- | --- | --- | --- |
|  | LASSO  (main analysis) | Ridge | Elastic Net  (α = 0.5) | Basic demographic | Basic demographic + severity |
| **Surgery Cost (Lower, %)** | 58.00 (57.95 - 58.05) | 58.03 (57.98 - 58.07) | 58.00 (57.95 - 58.05) | 57.96 (59.72 - 58.02) | 57.96 (57.92 - 58.02) |
| The reference group is low COC group (COC < 0.50 [median]). Effects of COC on surgery cost was estimated using a two-part model. For sensitivity analysis, we performed several models for predicting surgery costs for entire study population. With each predicted surgery costs, two-part model was performed. Each reduction in surgery costs was compared.  Basic demographic: age, gender, coverage  Severity: Frozen shoulder, shoulder impingement syndrome, Charlson Comorbidity Index | | | | | |

**Supplementary Table S3. Sensitivity analysis with tertiles standard**

| **COC** | **Surgery (OR)** | **Medical costs (lower)** |
| --- | --- | --- |
| **Low** | Reference | Reference |
| **Middle** | 0.40 (0.19 – 0.85) | 12.25 (4.90 – 19.05) |
| **High** | 0.51 (0.23 – 1.12) | 15.93 (8.64 – 22.63) |
| **P-trend** | 0.038 | <0.001 |
| The tertiles of COC were 0.40 and 0.60. The reference group is low COC group (COC < 0.40). Adjusted covariates are age in category, Gender, Coverage type, frozen shoulder, shoulder impingement syndrome, Charlson comorbidity index, primary provider type, and the total number of medical visits. | | |

**Supplementary Table S4. Sensitivity analysis added with 20% of S code as main diagnosis**

|  | Unadjusted | Adjusted |
| --- | --- | --- |
| **Surgery (OR)** | 0.29 (0.40 - 0.60) | 0.44 (0.21 - 0.89) |
| **Medical costs (lower)** |  |  |
| Direct medical cost (%) | 13.58 (7.02 - 19.68) | 14.33 (9.01 - 19.34) |
| Surgery Cost (%) | 55.67 (55.62 - 55.70) | |
| The reference group is low COC group (COC < 0.50 [median]). Effects of COC on surgery cost was estimated using a two-part model. Adjusted covariates are age in category, Gender, Coverage type, frozen shoulder, shoulder impingement syndrome, Charlson comorbidity index, primary provider type, and the total number of medical visits. | | |

**Supplementary Table S5. Results of hierarchical analysis with AIC and BIC**

|  | Model 1 | Model 2 | Model 3 | Model 4 | Model 5 |
| --- | --- | --- | --- | --- | --- |
| **Effects of COC in direct medical costs** | 8.09 (0.38 - 15.2) | 7.76 (0.07 - 14.86) | -1.80 (-10.05 - 5.83) | -3.24 (-11.62 - 4.52) | 14.09 (8.12 - 19.66) |
| **AIC** | 47933.4217 | 47909.4053 | 47739.0795 | 47729.1577 | 47093.162 |
| **Decrease in AIC** |  | 24.0164 | 170.3258 | 9.9218 | 635.9957 |
| **BIC** | 47947.7667 | 47947.5436 | 47804.4595 | 47799.986 | 47169.4387 |
| **Decrease in BIC** |  | 0.2231 | 143.0841 | 4.4735 | 630.5473 |
| The reference group is low COC group (COC < 0.50 [median]). The changes in effect, AIC, and BIC was observed with covariates included hierarchically. Model 1 : COC; Model 2 : COC + basic demographic (age, gender, coverage); Model 3 : COC + basic demographic + disease information (Frozen shoulder, Shoulder Impingement Syndrome, Charlson Comorbidity Index); Model 4 : COC + basic demographic + disease information + primary provider; Model 5 : COC + basic demographic + disease information + primary provider + total visit; | | | | | |
